# Risk Factors of Biochemical Failure in Locally Advanced Carcinoma Prostate Treated With Definitive External Beam Radiotherapy and Androgen Deprivation Therapy: Experience From Tertiary Care Center in North India

**DOI:** 10.7759/cureus.16895

**Published:** 2021-08-04

**Authors:** Manjinder Sidhu, Davinder Paul, Sandhya Sood, Kunal Jain, Jagdeep Singh, Ritu Aggarwal, Divyaanshi Sood

**Affiliations:** 1 Radiation Oncology, Dayanand Medical College & Hospital (DMCH) Cancer Center, Ludhiana, IND; 2 Medical Oncology, Dayanand Medical College & Hospital (DMCH) Cancer Center, Ludhiana, IND; 3 Oncology, Dayanand Medical College & Hospital (DMCH), Ludhiana, IND

**Keywords:** locally advanced prostate cancer, external beam radiotherapy, antiandrogen therapy, prognostic factors, biological failure

## Abstract

Background

Locally advanced prostate cancer (LACAP), despite external beam radiotherapy (EBRT) along with antiandrogen therapy (ADT) has risk of prostate-specific antigen (PSA) progression. Furthermore, number of studies have emphasized on different prognostic factors. The purpose of our study is to analyze risk factors for biochemical failure (BF) in these patients treated at our institute.

Methods

Our study is a single-institution retrospective observational done at a tertiary care center in North India. Between January 2018 and December 2020, we retrospectively identified 34 patients managed at our institute as per multidisciplinary board (MBD). Demographic, clinical, radiological, pathological and treatment-related parameters were assessed as potential risk factors. End-point of the study was to find significant risk factors for BF. Statistical analysis was done on SPSS, version 20 (IBM Corp., Armonk, NY).

Results

All eligible patients received EBRT with ADT as per institution policy. Mean follow-up period was 20 months during which two (5.9%) patients had BF at a mean of 30 months after EBRT. Four-year PSA-progression-free survival rate was 73%. On univariate analysis, prognostic factors associated with high risk of BF were Gleason score and clinical T stage.

Conclusion

In summary, prognostic factors for high risk of BF leading to clinical progression are Gleason score 9 or 10 and clinical T3b stage.

## Introduction

In India annual prostate cancer incidence rate ranges from 5.0 to 9.1 per 100,000/year [1], but also of all, 85% are detected late (stages III and IV). Furthermore, recommended treatment for locally advanced prostate cancer is external beam radiotherapy (EBRT) and antiandrogen therapy (ADT) as supported by the literature [2-4]. Nevertheless, a significant minority will eventually relapse [5,6]. It is important to note that the rise of prostate-specific antigen (PSA) above a defined threshold, which is called biochemical failure (BF), is usually the earliest harbinger of replaced disease after radiotherapy, and may manifest years prior to clinical recurrence [7].

Some of the high-risk factors in carcinoma prostate which have already been studied in various reports are: extent of the primary tumor (clinical stage), nodal status, degree of histological differentiation, zone of origin and serum PSA levels [8-11].

In this article, we retrospectively analyzed prognostic factors precisely leading to BF in our group of locally advanced prostate cancer (LACAP) prostate treated with standard EBRT and ADT.

## Materials and methods

Study design

Single institution cross-sectional retrospective analysis. The requirement for ethical approval was waived due to the retrospective study design.

Study place

The study was done at a tertiary care center in North India

Study period

Medical records of patients were collected from electronic medical record (EMR) available in Aria platform (version 16.1, CTSH [Cancer Treatment Services Hyderabad Pvt.], Ludhiana, Punjab) from January 2018 to December 2020. The last follow-up was May 2021.

Study population

Thirty-four biopsy-proven adenocarcinoma prostate referred to our department which met the criteria were included in the study. Inclusion criteria were men with clinical stage T3 to T4 adenocarcinoma prostate treated with definitive radiotherapy and ADT. Patients with positive lymph nodes were eligible if the involved nodes remained below the common iliac level. Early and Metastatic disease was excluded from the study.

Pre-treatment evaluation

All patients underwent physical examination, Karnofsky performance status evaluation, routine laboratory studies including serum prostate-specific antigen (PSA) and radiological workup included chest X-ray, bone scan or Positron Emission Tomography and Computed Tomography (PET-CT) scan (model: Discovery IQ, Make: GE Healthcare, Ludhiana, Punjab)

Procedure

Radiation Simulation, Planning and Delivery

All patients underwent immobilization in supine position on all-in-one immobilization system with both arms kept aside. Moreover, as per institution policy bladder filling protocol was followed, as well as it was ensured that rectum was empty during simulation. Contrast Computed tomography (CT) images (Discovery IQ, Make: GE Healthcare) were taken of lower abdomen and pelvis at 2.5-mm slice thickness. CT images were imported and contoured in Eclipse planning system version 16.1 (Varian Medical System, Palo Alto, CA).

For target delineation, we followed the Radiation Therapy Oncology Group (RTOG) contouring atlas for prostate cancer [12]. In addition, guidelines were also followed for pelvic nodal delineation [13]. Organ at risk delineated were: rectum, bladder, bowel and femoral heads. Entire rectum from the anal verge up to the recto-sigmoid junction was contoured [14-16]. Besides whole organ delineation, bladder wall and rectal wall were contoured using a 5-mm internal margin. Constraints for organs at risk were as follows: rectal wall: volume receiving 60 Gy ≤50% and volume receiving70 Gy ≤ 20%; bladder wall: volume receiving 65 Gy ≤ 50%; and femoral heads: volume (left, right) each receiving 50 Gy ≤10%. Treatment plans were generated using the TPS. All patients underwent rapid-arc planning. Patient underwent treatment on True Beam Linear accelerator (Varian Medical System, Palo Alto, CA) coupled with daily Cone-beam computed tomography systems (CBCT) for all patients. Daily CBCT of all patients were taken. Prescribed dose to PTV was 70 Gy in 35 fractions. Furthermore, pelvic lymph node received 50.4Gy in 28 fractions by SIB technique. Photon optimizer, version 13.7.16 was used for inverse optimization with 2.5mm optimization resolution. For calculation, anisotropic analytical algorithm (version 13.7.16) was used and the calculation grid was 2.5mm. Jaw-tracking option was selected to reduce the MLC leakage dose and inhomogeneity correction was applied for all plans. The isocenter was placed at center of PTV volume. The dose was prescribed such that >95% of the planning target volume received 100% of the prescribed dose. The rectal volume receiving >65 Gy and >50 Gy was limited to <17% and <35%, respectively. Likewise, bladder volume receiving >65 Gy and >40 Gy was limited to <25% and <50%, respectively. Furthermore, small bowel (peritoneal cavity) constraints were V45Gy <195cc [17].

Androgen Deprivation Therapy

Androgen deprivation was used at the discretion of the physician. All patients received ADT before, during and after the planned course of radiotherapy. ADT consisted primarily of an oral antiandrogen and luteinizing hormone-releasing hormone agonist administered as subcutaneously depot injections.

Follow-up

Generally, follow-up examinations were performed initially at every three months after radiotherapy treatment during the first year, and subsequently at six-month intervals with serial PSA determination and physician-performed digital rectal examination at each visit. BF was defined as a rise of 2 ng/mL or above the nadir PSA after EBRT with or without hormonal therapy [18]. The interval to BF (IBF) was defined as the time from completion of radiotherapy to BF. Distant metastasis (DM) was defined as metastasis in the bones, visceral organs, or lymph nodes outside of the pelvis. Imaging coupled with biopsy was done as per the discretion of the treating physician.

Study outcomes

The endpoint was to evaluate significant prognostic risk factors for BF. PSA-progression-free survival rate was defined as the time from date of end of treatment to date of event defined as first documented BF as per phoenix definition. For the purposes of the current analysis, regional metastasis is defined as clinical or radiographic evidence of involvement of the pelvic lymphatics by the tumour beyond completion of adjuvant ADT. Distant metastases are defined as clinical or radiographic evidence of disease beyond the pelvis during follow-up.

Statistical analysis

All data were analysed using SPSS Statistics for Windows, Version 20.0 (IBM Corp., Armonk, NY). Generalized linear modelling was done for univariate analysis to establish the association of prognostic factors for the locoregional control. Continuous variables were dichotomized according to their mean values or split into subgroups depending on their clinical significance. Pearson’s chi-square test was done for categorical variables. Multivariate analysis by logistic regression was not done in view of insufficient sample size. Survival curves were estimated using the K-M method. For all practical purposes, a p-value of 0.05 or less was considered significant.

## Results

All 34 patients were included for analysis. Baseline clinical, pathologic, and treatment characteristics are detailed in Table 1.

**Table 1:** Characteristics of the study population. PSA: prostate-specific antigen.

Characteristic	Number of patients (%)
Mean age(years)	68
Mean baseline PSA (ng/mL)	35.3
Gleason group grade	
1	6(17.6%)
2	7(20.6%)
3	11(32.4%)
4	6(17.6%)
5	4(11.8%)
Gleason score	
<=6	7(20.6%)
7	16(47.1%)
8	6(17.6%)
9 or 10	5(14.7%)
Gleason pattern	
≤3+3	7(20.6%)
3+4	7(20.6%)
4+3	9(26.5%)
4+4,3+5,5+3	6(17.6%)
4+5,5+4,5+5	5(14.7%)
Clinical T stage	
T1	2(5.9%)
T2b	2(5.9%)
T2c	1(2.9%)
T3a	3(8.8%)
T3b	17(50%)
T4	9(26.5%)
Clinical N stage	
N0	21(61.8%)
N1	13(38.2%)
Clinical stage	
IIIA	4(11.8%)
IIIB	13(38.2%)
IIIC	4(11.8%)
IVA	13(38.2%)
Mean radiotherapy dose (Gy)	70
Mean number of fractions	35
Mean PSA nadir (ng/mL)	0.27

Overall, the median age of diagnosis was 68 years (range: 50-76 years) with mean PSA at time of diagnosis 35.3 ng/mL. Most common Gleason group grade was 3. Similarly, Gleason score 7 was commonly seen. Likewise, among Gleason patterns, 4+3 was slightly more prevalent compared to others. In addition, clinical T3b and N0 represented 50% and 61.8% cases respectively. Furthermore, among these locally advanced cases stage IIIB and IVA constituting 76.4% (N=26) of cases. Mean size of prostate tumor was 4cm (range 1-6.8cm) with peripheral zone being the most common site in 67.3% (N=23).

All patients received neoadjuvant and adjuvant ADT with a mean of five and 25 months prior to start and completion of radiotherapy respectively coupled with concurrent ADT in all cases. Commonly used hormonal injection was Lupride in 38.2% (N=13) patients. Radiotherapy was delivered by rapid arc technology in all with mean of 70Gy in 35 fractions.

BF and univariate analysis

Mean follow-up from the end of radiotherapy was 20 months. Two (5.9%) patients had BF according to Phoenix definition at mean IBF of 30 months after radiotherapy during the study follow-up. For the subset of patients with BF, the median time from BF to clinically detected metastasis was eight months and out of these one had visceral and other had local recurrence which were confirmed by PET-CT and biopsy respectively. Variable explored for relationship with BF are detailed in Table 2.

**Table 2:** Variables explored for relationship with biological failure. BF: biochemical failure; PSA: prostate-specific antigen; PNI: perineural invasion.

Variables	Total	BF	No BF	Sig.
Age				
<=68	18(52.9%)	1(50%)	17(53.1%)	0.727
>68	16(47.1%)	1(50%)	15(46.9%)	
Baseline PSA				
<=20	14(53.8%)	1(50%)	13(54.2%)	0.720
>20	12(46.2%)	1(50%)	11(45.8%)	
Gleason score				
<=6	7(20.6%)	0(0%)	7(21.9%)	
7	16(47.1%)	0(0%)	16(50%)	
8	6(17.6%)	0(0%)	6(18.8%)	
9-10	5(14.7%)	2(100%)	3(9.4%)	0.006
PNI				
Yes	11(32.4%)	0(0%)	11(34.4%)	
No	23(67.6%)	2(100%)	21(65.6%)	0.451
Size of tumor				
<=4cm	22(64.7%)	2(100%)	20(65.5%)	
>4cm	12(35.3%)	0(0%)	12(37.5%)	0.421
cT stage				
T1	2(5.9%)	0(0%)	2(6.2%)	
T2a	2(5.9%)	0(0%)	2(6.2%)	
T2c	1(2.9%)	1(50%)	0(0%)	
T3a	3(8.8%)	0(0%)	3(9.4%)	0.004
T3b	17(50%)	1(50%)	16(50%)	
T4	9(26.5%)	0(0%)	9(28.1%)	
c N stage				
N0	21(61.8%)	1(50%)	20(65.5%)	
N1	13(38.2%)	1(50%)	12(37.5%)	0.626

**Figure 1:**
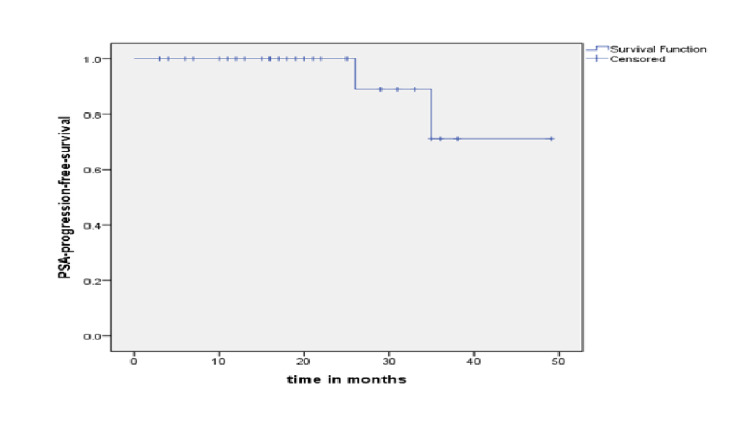
Prostate-specific antigen progression-free survival rate.

On univariate analysis by GLIM, the following prognostic factors were associated with high risk of BF, Gleason score X2 (3)=12.32, p=0.006 and clinical T staging X2 (5)=17.0, p=0.004. Overall, four-year PSA-progression-free survival rate of the cohort was 73% (Figure 1).

## Discussion

Our results showed that unfavourable risk factors for early BF in locally advanced carcinoma prostate treated with definitive radiotherapy with ADT are pre-treatment GS 9 or 10 and higher T stage. In these subsets of patient, cases with T3b disease progressed earlier to metastatic disease.

In our study, more than 70% of patients were of high-risk disease, mainly due to the high initial PSA and Gleason grades at the time of diagnosis. Such finding in our patient characteristics is similar to other reports stating that Asians have a higher tendency for having high-risk disease when matched with the Western population [19,20]. Furthermore, these patients are treated at our hospital with RT compared to radical prostatectomy, based on rationale that the outcome is similar when patients are matched by stage and tumour grade [21]. Four-year biochemical free survival rate in our study is 73%. Similarly, for comparison, a compiled report of 34 Japanese institutions reported a 5-year BFS of 71.9% [22]. It is important to note that patient characteristic and median radiation dose was similar in our study and above Japanese study. In addition, more than 80% of this series patients were treated with rotation technique similar to our study in which we treated all patients with rapid arc rotational technique. Also, note that all our patients received long-term ADT with mean of 25 months duration which is now standard recommendation for locally advanced carcinoma prostate [23]. Likewise, all our patients received pelvic nodal irradiation despite presently there is a great controversy regarding the effectiveness of elective pelvic radiotherapy in patients with high-risk prostate cancer. On the other hand, the analysis of a recent randomized trial has demonstrated that pelvic irradiation is associated with an improvement in the progression-free survival when neoadjuvant HT is used in conjunction with EBRT [24]. Significant prognostic factors for biochemical recurrence in our study were higher GS and advanced T stage which is supported by various other studies [21,25].

Taken together our findings and findings of previous studies [26] point towards a higher risk of biochemical failure and subsequent clinical progression after EBRT and ADT in locally advanced carcinoma prostate specifically those with GS 9 or 10 and or advanced T stage.

Our study has two major limitations. Firstly, we were not able to do multivariate logistic regression for accurate analysis of predictive factors in view of less sample size. Nevertheless, we were able to address prognostic factors by univariate analysis. Secondly, our analysis was retrospective in nature. Though all data elements were prospectively collected and follow-up was done periodically, there were a lot of lost cases excluded from the analysis. Consequently, the remaining sample eligible for analysis may result in overestimation or underestimation of survival.

## Conclusions

Among various demographic, clinical, radiological, pathological and treatment-related parameters explored for relationship with biological failure in our subset of locally advanced carcinoma prostate patients which were treated by external beam radiotherapy and antiandrogen therapy, the most significant were: pre-treatment GS 9 or 10 and higher clinical T stage, precisely T3b. Our results provide potentially clinical useful predictive tools for physicians and patients for locally advanced prostate cancer that necessitates additional measures in these subsets of cases.
